# Snakebites caused by the genera *Bothrops* and *Lachesis* in the Brazilian Amazon: a study of factors associated with severe cases and death

**DOI:** 10.1590/0037-8682-0558-2021

**Published:** 2022-07-25

**Authors:** Samara Freire Valente Magalhães, Henry Maia Peixoto, Lúcia Rolim Santana de Freitas, Wuelton Marcelo Monteiro, Maria Regina Fernandes de Oliveira

**Affiliations:** 1 Universidade de Brasília, Núcleo de Medicina Tropical, Brasília, DF, Brasil.; 2 Conselho Nacional de Desenvolvimento Científico e Tecnológico, Instituto de Avaliação de Tecnologias em Saúde, Porto Alegre, RS. Brasil; 3 Ministério da Saúde, Secretaria de Vigilância em Saúde, Brasília, DF, Brasil.; 4 Fundação de Medicina Tropical Dr. Heitor Vieira Dourado, Manaus, AM, Brasil.; 5 Universidade do Estado do Amazonas, Manaus, AM, Brasil.

**Keywords:** Epidemiology, Snakebites, Risk factors, Death

## Abstract

**Background:**

Snakebites are a major problem in developing countries owing to their high morbidity rates, severity, and sequelae. In Brazil, most cases of envenomation are caused by *Bothrops* and *Lachesis* snakes. The present study aimed to evaluate variables associated with death, systemic complications, and amputations in victims of envenomation due to *Bothrops* or *Lachesis* snake.

**Methods:**

An analytical epidemiological study was performed with data from the Notifiable Diseases Information *System* (Sistema de Informação de Agravos de Notificação [*SINAN]*) relating to the Brazilian Amazon for the period 2010-2015. A hierarchical Poisson regression analysis was performed with three aspects, namely, individual characteristics, sociodemographic characteristics, and clinical conditions.

**Results:**

The following associations were observed: i) advanced age (>65 *years*), sepsis, renal failure, and hemorrhagic manifestations were related to two outcomes - death due to snakebite and death from all causes; ii) more advanced age (≥46 *years*), time to health care longer than 6 hours, renal and hemorrhagic manifestations, and region of occurrence being rural areas were associated with systemic complications; and iii) children (up to 12 years old) were associated with amputations.

**Conclusions:**

Knowledge of the characteristics associated with severe outcomes in snakebites may help identify patients who will require more intensive care or longer follow-up and may provide the physician with counseling rationale for their possible prognosis.

## INTRODUCTION

Snakebites are a relevant public health issue worldwide, especially in the tropics, where they are a regular problem that can lead to morbidity and death^1^. It is estimated that approximately 81,000 to 138,000 people globally die from snakebites every year. In 2017, snakebites were reinstated to the list of neglected tropical diseases by the World Health Organization (WHO)^1^ ^-^ ^4^, which is a clear indication that this is a relevant issue and that the WHO aims to bring it under control.

The WHO has been working on strategies to prevent and control snakebite envenomation, with an aim to reduce by half the number of deaths and disability cases due to snakebite envenomation over the following years through a program that targets affected communities and their health systems^5^.

Envenomation is a process wherein the toxins are introduced into the body through the snake’s fangs, and these toxins produce effects in the areas around the bite, which eventually produce systemic effects. Very often, these effects can have a serious impact. Envenomation can require a range of healthcare services, such as surgery and intensive care for severe cases, as well as rehabilitation^6^ ^,^ ^7^. People who are mainly affected include men and young adults in the poor rural communities of developing countries^8^ ^-^ ^10^. 

The clinical consequences of a snakebite can vary depending on the development of the patient’s condition, and the potential complications may be serious. Many of the complications can cause permanent sequelae and impose cost burden to the patient, society, and the healthcare system. A recent study estimated the costs of snakebite-related illness and reported that almost US $8 million was spent for snakebite treatment in the Brazilian Amazon in 2015^11^.

Snakebites in Brazil must be notified to the official health service register. Notifiable diseases can include various health conditions that, upon detection, are required to be reported to public health authorities using the Notifiable Diseases Information System (Sistema de Informação de Agravos de Notificação [SINAN]) in Brazil. This system provides information for the analysis of morbidity and assists decision-making at all levels of government, namely, municipal, state, or federal. 

Most cases of snakebites reported in the Brazilian Amazon are *caused* by the *genus Bothrops*, accounting for 87% of all snakebite cases and for the highest proportion of deaths and amputations^12^. The *genus Lachesis* is the second most common cause of snakebites, accounting for approximately 8% of all envenomation cases that occurred during 2010-2015^12^. When comparing the envenomations caused by *Bothrops* and *Lachesis* snakes, both envenomations had similar clinical features such as inflammation, coagulation, and hemorrhage and similar symptoms such as edema, pain, local and systemic hemorrhage, necrosis, and acute renal failure.

The Amazon region of Brazil has an area of 5.2 million square kilometers (61% of the Brazilian territory) but a demographic density of only 3.67 inhabitants/km^2^. Nonetheless, the region has faced a demographic expansion that has led to a population growth in the capital cities, although a notable proportion of the population still lives in rural areas near the jungle^13^. The hallmarks of this region include difficulty in access to and within the region because there are only a few highways. Certainly, snakebites may be extremely common in this scenario. In Brazil, the public healthcare system (Sistema Único de Saúde) has been providing free access to all levels of healthcare for over three decades, which has contributed to improving health indicators and has increased the life expectancy of the county’s population.

This study aimed to analyze the factors associated with death, systemic complications, and amputation reported in cases of snakebite (particularly genera *Bothrops* and *Lachesis*) that occurred in the Brazilian Amazon during 2010-2015.

## METHODS

An analytical epidemiological study was conducted among 62,591 cases registered on the Notifiable Diseases Information System (SINAN) in the Brazilian Amazon. The cases involving envenomation by snakes of the genera *Bothrops* and *Lachesis* that occurred during 2010-2015 were assessed. Cases of missing data were excluded.

The following four outcomes were evaluated: i) deaths caused by snakebite: death, wherein the cause registered was snakebite; ii) death from all causes: death caused by snakebite plus death by any cause registered as being any cause other than snakebite - for example, any clinical complications; iii) presence of systemic complications - a set of clinical conditions that encompassed shock, acute kidney failure (AKF), sepsis, and acute pulmonary edema; and iv) amputation. 

The associations between the recorded outcomes and independent variables listed on SINAN were examined using a hierarchical Poisson regression analysis. The three levels of analysis and their respective independent variables were as follows: (1) individual characteristics (distal level) - i) sex (female or male); ii) ethnicity (black, mixed, or white); iii) pregnant (yes or no); iv) age range (0-15, 16-45, 46-65, and >65 years old) and up to 12 years old when compared with other age groups, for amputation analysis; (2) Sociodemographic variables (intermediate level) - (i) education (complete primary school education, higher education, and other categories); (ii) location (rural or other areas; the variable “other areas” encompasses urban and suburban areas); (iii) work-related accidents (yes or no); (3) clinical conditions (proximal level) - (i) location of the bite (upper or lower body); (ii) time until the patient received health care (up to 6 h and over 6 h); (iii) secondary infection (yes or no); (iv) sepsis (yes or no); (v) kidney disorder (yes or no), and (vi) evidence of hemorrhage (yes or no). The definitions of independent variables and outcomes are presented in Table 1. 

**TABLE 1:** Definitions of independent variables and outcomes.

Variables	Definitions
Local complications	Delimited in a specific location on the body, such as edema, ecchymosis, necrosis, and others
Systemic complications	Those affecting some of the internal organs
Systemic hemorrhage complications	Gingival bleeding and other bleeding
Systemic vagal manifestations	Vomiting and diarrhea
Systemic hemolytic manifestations	Myalgia, anemia, dark urine
Systemic renal manifestations	Oliguria and anuria
Deaths caused by snakebite	Death, wherein the cause registered was snakebite
Deaths from all causes	Death caused by snakebite plus death due to any cause registered as being other than snakebites - e.g., any clinical complications

The relative risk (RR) and their respective confidence intervals (95% CIs) were estimated. Bivariate and multivariate analyses were constructed, with a statistical significance level (p-value) of ≤0.20 for variable selection to the multivariate model. Multivariate analysis was conducted using a hierarchical approach, and the model incorporated the following three levels: the distal level, which included individual variables; the intermediate level, which included demographic variables; and the proximal level, which included clinical variables. The backward method was used on each of the hierarchical levels to select the independent variables. The Akaike information criteria were used to select the models. Standard graphs of Poisson probability distribution were used to assess the goodness-of-fit to the model. This final analysis was completed at a critical decision level of 5% using statistical software (STATA^®^, version 15.0).

### Ethics approval

The research project was submitted to the Ethics Committee of the Faculdade de Medicina da Universidade de Brasília and was approved under assessment number 1652440 on July 28, 2016. 

## RESULTS

Most snakebites were caused by snakes of the genus *Bothrops*, accounting for 57,374 cases (91.7%), and snakebites from the genus *Lachesis* accounted for 5,217 cases (8.3%). Among 315 recorded deaths (0.50%), 291 (0.46% of the sample) were registered as deaths caused by snakebite. Systemic complications occurred in 649 patients (1.0%), and amputation occurred in 57 cases (0.09%). 

Bivariate analysis of deaths due to snakebites showed that level of schooling, location - rural area, and age range of 46-65 years and above 65 years were the only variables from intermediate and distal levels, which were statistically relevant (Table 2). At the proximal level, among the clinical variables, there were significant associations with all the variables, except the snakebite region on the body. In the multivariable model, presence of sepsis (RR: 3.83; 95% CI: 2.50-5.87), AKF (RR: 3.89; 95% CI: 2.17-6.99), and evidence of hemorrhage (RR: 1.97; 95% CI: 1.30-2.98) as well as the age range of 46-65 years (RR: 1.81; 95% CI: 1.16-2.82) and above 65 years (RR: 3.01; 95% CI: 1.71-5.30) were all positively associated with deaths due to snakebite (Table 2). 

**TABLE 2:** Factors associated with deaths caused by snakebite by the genera *Bothrops* and *Lachesis* in Brazilian Amazon during 2010-2015.

Variables*	Death by snakebite	No outcome	Crude RR (95% CI)	p-value	Adjusted RR (95% CI)	p-value
	N (%)	N (%)				
**Proximal - clinical conditions**						
**Location of the bite**						
Lower body	250 (0.56%)	44,485 (99.44%)	1.18 (0.85-1.65)	0.326	-	-
Upper body	40 (0.47%)	8,418 (99.52%)				
**Time between bite and patient receiving care**						
Over 6 h	101 (0.88%)	11,330 (99.12%)	2.10 (1.65-2.70)	<0.0005	1.36 (0.86-2.14)	0.182
Up to 6 h	165 (0.42%)	39,184 (99.58%)				
**Sepsis**						
Yes	34 (46.58%)	39 (53.42%)	3.73 (2.65-5.25)	<0.0005	3.83 (2.50-5.87)	<0.0005
No	60 (12.47%)	421 (87.53%)				
**Acute kidney failure**						
Yes	70 (26.32%)	196 (73.68%)	2.95 (1.95-4.46)	<0.0005	3.89 (2.17-6.99)	<0.0005
No	27 (8.91%)	276 (91.09%)				
**Evidence of hemorrhage**						
Yes	54 (1.89%)	2,796 (98.11%)	1.53 (1.07-2.18)	0.018	1.97 (1.30-2.98)	0.001
No	68 (1.24%)	5,432 (98.76%)				
**Kidney disorder**						
Yes	65 (6.86%)	882 (93.14%)	8.14 (6.08-12.36)	<0.0005	1.34 (0.84-2.14)	0.212
No	62 (0.84%)	7,290 (99.16%)				
**Intermediate - Socio-demographic variables**						
Level of schooling						
Failed to complete up to 4^th^ grade	106 (0.66%)	15,958 (99.34%)	1.44 (1.09-1.90)	0.01	1.19 (0.88-1.59)	0.252
Other education levels	94 (0.46%)	20,386 (99.54%)				
**Work-related accident**						
Yes	119 (0.62%)	18,984 (99.38%)	1.16 (0.91-1.48)	0.220	-	-
No	152 (0.54%)	28,188 (99.46%)				
**Location (area)**						
Rural area	264 (0.57%)	45,806 (99.43%)	1.68 (1.09-2.57)	0.018	1.56 (0.92-2.64)	0.09
Other areas	23 (0.34%)	6,694 (99.66%)				
**Distal** - **Individual variables**						
**Sex**						
Female	60 (0.53%)	11,335 (99.47%)	0.96 (0.72-1.28)	0.786	-	-
Male	231 (0.55%)	41,954 (99.45%)				
**Ethnicity**						
Mixed race	192 (0.51%)	37,759 (99.49%)	0.77 (0.54-1.14)	0.456	-	-
Not mixed race	87 (0.67%)	12,910 (99.33%)				
**Age groups**						
0-15	47 (0.42%)	11,271 (99.58%)	1	-	1	-
16-45	126 (0.43%)	29,324 (99.57%)	1.03 (0.74-1.44)	0.861	0.99 (0.65-1.50)	0.952
46-65	85 (0.80%)	10,498 (99.20%)	1.94 (1.36-2.77)	<0.0005	1.81 (1.16-2.82)	0.009
>65	33 (1.48%)	2,203 (98.52%)	3.59 (2.30-5.62)	<0.0005	3.01 (1.71-5.30)	<0.0005

**RR:** relative risk; 95% CI: 95% confidence interval. *Only valid cases.

Very similar results were observed for death from all causes as the outcome, with the only difference in the 46-65 age range, which did not appear to be associated with the outcome (Table 3). 

**TABLE 3:** Factors associated with deaths from all causes in victims of snakebites caused by the genera *Bothrops* and *Lachesis* in the Brazilian Amazon, during 2010-2015.

Variable*	Death from all causes	No outcome	Crude RR (95% CI)	p-value	Adjusted RR (95% CI)	p-value
	N (%)	N (%)				
**Proximal - clinical conditions**						
**Location of the bite**						
Lower body	270 (0.60%)	44,485 (99.40%)	1.16 (0.84-1.60)	0.36	-	-
Upper body	44 (0.52%)	8,418 (99.48%)				
**Time between bite and patient receiving care**						
Up to 6 h	107 (0.94%)	11,330 (99.06%)	2.05 (1.62-2.61)	<0.0005	1.34 (0.86-2.08)	0.194
Over 6 h	180 (0.46%)	39,184 (99.54%)				
**Sepsis**						
Yes	35 (47.30%)	39 (52.70%)	3.63 (2.60-5.07)	<0.0005	3.72 (2.44-5.68)	<0.0005
No	63 (13.02%)	421 (86.98%)				
**Acute kidney failure**						
Yes	73 (27.14%)	196 (72.86%)	2.95 (1.97-4.41)	<0.0005	2.91 (1.62-5.22)	<0.0005
No	28 (9.21%)	276 (90.79%)				
**Evidence of hemorrhage**						
Yes	56 (1.96%)	2,796 (98.04%)	1.44 (1.02-2.03)	0.037	1.70 (1.13-2.55)	0.011
No	75 (1.36%)	5,432 (98.64%)				
**Kidney disorder**						
Yes	68 (7.16%)	882 (92.84%)	7.62 (5.49-10.59)	<0.0005	1.37 (0.87-2.15)	0.170
No	69 (0.94%)	7,290 (99.06%)				
**Intermediate - Socio-demographic variables**						
**Level of schooling**						
Failed to complete up to 4^th^ grade	115 (0.72%)	15,958 (99.28%)	1.48 (1.13-1.94)	0.004	1.23 (0.92-1.64)	0.158
Other education levels	99 (0.48%)	20,386 (99.52%)				
Work-related accident						
Yes	129 (0.67%)	18,984 (99.33%)	1.19 (0.94-1.50)	0.143	0.97 (0.72-1.30)	0.851
No	161 (0.57%)	28,188 (99.43%)				
**Location (area)**						
Rural area	286 (0.62%)	45,806 (99.38%)	1.67 (1.11-2.52)	0.014	1.58 (0.93-2.68)	0.089
Other areas	25 (0.37%)	6,694 (99.63%)				
**Distal - individual variables**						
**Sex**						
Female	63 (0.55%)	11,335 (99.45%)	0.92 (0.70-1.22)	0.58	-	-
Male	252 (0.60%)	41,954 (99.40%)				
**Ethnicity**						
Mixed race	210 (0.55%)	37,759 (99.45%)	0.79 (0.53-1.50)	0.52	-	-
Not mixed race	91 (0.70%)	12,910 (99.30%)				
**Age groups, years**						
0-15	50 (0.44%)	11,271 (99.56%)	1	-	1	-
16-45	131 (0.44%)	29,324 (99.56%)	1.01 (0.73-1.39)	0.97	1.21 (0.58-2.50)	0.615
46-65	96 (0.91%)	10,498 (99.09%)	2.06 (1.46-2.88)	<0.0005	1.70 (0.88-3.59)	0.140
>65	38 (1.70%)	2,203 (98.30%)	3.89 (2.54-5.94)	<0.0005	3.76 (1.74-8.19)	0.001

**RR:** relative risk; 95% CI: 95% confidence interval. *Only valid cases.

When considering systemic complications, the results of bivariate analysis of the proximal level showed that all variables had association, except for the physical location of the bite. Regarding the intermediate variables, the location - snakebite occurring in the rural area - and level of schooling were associated with the outcome, and among the distal variables, only the age group was associated with systemic complications. The multivariate model demonstrated a significant association between systemic complications and the following variables: presence of kidney disorders (RR: 6.76; 95% CI: 5.60-8.17), time between the bite and the care provided to the victim being greater than 6 h (RR: 2.47; 95% CI: 2.03-3.00), patients presenting hemorrhage (RR: 1.40; 95% CI: 1.16-1.69), snakebite occurrence being a rural area (RR: 1.89; 95% CI: 1.26-2.83*)*, age groups of 46-65 years old (RR: 1.38; 95% CI: 1.01-1.89) and above 65 years (RR: 2.15; 95% CI: 1.49-3.10) (Table 4). 

**TABLE 4:** Factors associated with systemic complications in victims of snakebites caused by the genera Bothrops and Lachesis in the Brazilian Amazon, during 2010-2015.

Variable*	Systemic complications	No outcome	Crude RR (95% CI)	p-value	Adjusted RR (95% CI)	p-value
	N (%)	N (%)				
**Proximal - clinical conditions**						
**Location of the bite**						
Lower body	540 (1.25%)	42,707 (98.75%)	0.99 (0.80-1.23)	0.95	-	-
Upper body	104 (1.26%)	8,169 (98.74%)				
**Time between bite and patient receiving care**						
Up to 6 h	322 (2.91%)	10,752 (97.09%)	3.72 (3.18-4.34)	<0.0005	2.47 (2.03-3.00)	<0.0005
Over 6 h	301 (0.78%)	38,127 (99.22%)				
**Evidence of hemorrhage**						
Yes	180 (6.55%)	2,569 (93.45%)	1.34 (1.11-1.61)	0.002	1.40 (1.16-1.69)	<0.0005
No	260 (4.89%)	5,061 (95.11%)				
**Kidney disorders**						
Yes	224 (24.22%)	701 (75.78%)	7.93 (6.67-9.43)	<0.0005	6.76 (5.60-8.17)	<0.0005
No	217 (3.05%)	6,890 (96.95%)				
**Intermediate** - **sociodemographic variables**						
**Level of schooling**						
Failed to complete up to 4^th^ grade	226 (1.41%)	15,827 (98.59%)	1.25 (1.04-1.51)	0.016	1.07 (0.88-1.30)	0.462
Other education levels	227 (1.12%)	19,985 (98.88%)				
**Location (area)**						
Rural area	598 (1.34%)	44,184 (98.66%)	1.90 (1.40-2.59)	<0.0005	1.89 (1.26-2.83)	0.002
Other areas	45 (0.70%)	6,345 (99.30%)				
**Distal - Individual variables**						
**Sex**						
Female	124 (1.13%)	10,856 (98.87%)	0.88 (0.72-1.07)	0.194	0.88 (0.73-1.08)	0.229
Male	525 (1.28%)	40,343 (98.72%)				
Ethnicity						
Mixed race	467 (1.24%)	37,120 (98.76%)	1.28 (0.95-1.73)	0.108	1.07 (0.76-1.53)	0.680
Not mixed race	168 (1.33%)	12,497 (98.67%)				
**Age groups**						
0-15	119 (1.09%)	10,751 (98.91%)	1	-	1	-
16-45	328 (1.15%)	28,155 (98.85%)	1.05 (0.85-1.30)	0.634	1.06 (0.85-1.31)	0.612
46-65	153 (1.48%)	10,155 (98.52%)	1.36 (1.07-1.73)	0.012	1,38 (1.01-1.89)	0.039
>65	49 (2.24%)	2,143 (97.76%)	2.06 (1.48-2.89)	<0.0005	2.15 (1.49-3.10)	<0.0005

**RR:** relative risk; 95% CI: 95% confidence interval. *Only valid cases

The results of bivariate analysis indicated that the age group of up to 12 years among children was significantly associated with amputation (Table 5). No other variable presented a significant association, although evidence of hemorrhage did show probability close to the decision point (Table 5). Based on the results of multivariate analysis, only age group, specifically up to 12 years old among children, was still associated with the outcome (RR: 2.24; 95% CI: 1.24-4.06) (Table 5).

**TABLE 5:** Factors associated with amputation due to snakebites caused by the genera Bothrops and Lachesis, in the Brazilian Amazon, during 2010-2015.

Variables*	Amputation	No outcome	Crude RR (95% CI)	p- value	Adjusted RR (95% CI)	p- value
	N (%)	N (%)				
**Proximal - clinical conditions**						
**Location of the bite**						
Lower body	40 (1.56%)	2,516 (98.44%)	0.62 (0.33-1.13)	0.12	1.13 (0.32-3.96)	0.85
Upper body	14 (2.53%)	540 (97.47%)				
**Time between bite and patient receiving care**						
Up to 6 h	24 (2.39%)	979 (97.61%)	1.55 (0.91-2.64)	0.1	1.15 (0.44-2.95)	0.77
Over 6 h	30 (1.54%)	1,918 (98.46%)				
**Acute kidney failure**						
Yes	10 (6.29%)	149 (93.71%)	1.04 (0.31-3.44)	0.95	-	-
No	4 (6.06%)	62 (93.94%)				
**Evidence of hemorrhage**						
Yes	11 (2.63%)	408 (97.37%)	2.39 (0.93-6.11)	0.07	2.35 (0.9-6.15)	0.08
No	7 (1.10%)	630 (98.90%)				
**Kidney disorders**						
Yes	5 (2.45%)	199 (97.55%)	1.61 (0.57-4.57)	0.37	-	-
No	13 (1.53%)	834 (98.47%)				
**Intermediate - sociodemographic variables**						
**Level of schooling**						
Completed primary education	31 (1.62%)	1,884 (98.38%)	0.91 (0.47-1.76)	0.79	-	-
Other education levels	5 (2.08%)	235 (97.92%)				
**Work-related accident**						
Yes	22 (1.54%)	1,403 (98.46%)	0.82 (0.47-1.44)	0.49	-	-
No	28 (1.87%)	1,470 (98.13%)				
**Location (area)**						
Rural district	52 (1.84%)	2,768 (98.16%)	2.43 (0.59-9.90)	0.21	-	-
Other areas	2 (0.76%)	261 (99.24%)				
**Distal - individual variables**						
**Sex**						
Female	8 (1.30%)	608 (98.70%)	0.70 (0.33-1.49)	0.36	-	-
Male	46 (1.84%)	2,454 (98.16%)				
**Ethnicity**						
Mixed race	38 (1.74%)	2,146 (98.26%)	1.03 (0.40-2.50)	0.92	-	-
Not mixed race	14 (1.69%)	814 (98.31%)				
**Age groups**						
Children up to 12 years old	16 (3.21%)	483 (96.79%)	2.24 (1.23-3.93)	0.008	2.24 (1.24-4.06)	0.007
Other groups	35 (1.45%)	2,376 (98.55%)				

**RR:** relative risk; 95% CI: 95% confidence interval. *Only valid cases.

## DISCUSSION

Notification data in Brazil are the best source of public information currently available regarding snakebites. It is the only regular data source on their occurrence, mainly because there is little incentive to conduct research on the topic; thus, this topic is not studied to a great degree, either in Brazil or in the rest of the world^14^.

Snakebites can cause potentially severe outcomes. There were 315 deaths (0.50%) and 57 amputations (0.09%) in this studied series, which could be averted. Some variables were associated with more than one outcome. The most relevant factors that were associated with the study outcomes are discussed below. 

The time elapsed until receiving healthcare is an extremely important factor and greatly affects the patient’s clinical condition and the outcome. The shorter the time between the bite and the adequate care provided, the better the prognosis^10^ ^.^ Time to treatment was classified as early when less than 6 hours or as delayed when beyond 6 hours and was strongly associated with systemic complications. Time to receive medical assistance being beyond 6 hours was an independent risk factor for severity^10^. The results corroborate recent studies that demonstrated that a distance greater than 300 km between the location where the snakebite occurred and the city of Manaus was a factor associated with cases of mortality due to snakebite envenomation that occurred in this region^15^. In Nepal, running a campaign to raise people’s awareness of the importance of a quick response to a bite, coupled with providing an incentive to motorcycle owners to provide transportation in cases of snakebites, reduced the case fatality rate from 10.5% to 0.5%^16^. This would be an interesting option for Brazilian health officials to consider and likewise assess the potential impact. In healthcare services for rural riverside populations in the Brazilian Amazon, fluvial mobile units are an advancement for improving access to healthcare from user’s and professional’s perspectives^17^. 

It should be noted the variable “rural area” was another factor associated with systemic complications, which may be due to the difficulties in transporting people to health centers where antivenom is available and, in some cases, this may involve more than one type of transport^15^ ^,^ ^16^. 

It is also worth noting that rural workers, in general, have a low income and a low education level, and studies have shown that there is a strong connection between poverty and snakebites^4^ ^,^ ^18^ ^-^ ^19^. One study conducted in 2019 stated that snake envenomation is not only a consequence of rural poverty but also a cause of it. The authors believed it to be probably the most neglected condition, with the highest mortality and morbidity rates among all neglected tropical diseases in the world^20^. Although education levels are associated with the degree of vulnerability and the illnesses people experience, there was no association between this variable and the study outcomes.

Another factor observed from the analysis was that more advanced age was associated with deaths and systemic complications. This result can be explained by the likely additional risk factors and the natural changes in the body due to aging.

Amputation was a rare event in the sample. This may indicate better wound healing in most cases, which suggests that it has been properly treated after the snakebite. The low frequency of the event could explain a single connection between the variables, but there are insufficient numbers for quantitative exploration. The age range variable shows that children aged up to 12 years were 2.24 times more likely to experience amputations than victims in the other age groups. This finding reinforces the importance of adequate care, in particular, the careful and early use of fasciotomy, which can reduce the complications observed in children who have a snakebite^21^. There is little information in the literature reporting the occurrence of snakebites in patients up to 12 years of age. The low incidence is probably related to the differences between the habits of children and snakes, e.g., a child is less likely to be exposed to closed forest environments^22^ ^,^ ^23^. Additionally, children are not injected with less venom compared with adults. Therefore, the concentration of free fraction in the target organs is higher in children than in adults, which may explain this finding^22^ ^,^ ^23^; as such, this could result in local and functional anatomical sequelae, and this often leads to tissue necrosis and secondary infection, which tends to result in amputation^23^. 

Among the cases involving systemic complications, AKF was the most frequent, both in general and in the context of patients who died. Evidence suggests that AKF is generally the leading cause of death in snakebite cases^24^. 

The analysis of the variables associated with deaths, regardless of whether due to snakebites or all causes, showed very similar results. As there is no access to other information regarding deaths from other causes and considering clinical characteristics of snakebites being complex and extensive, the number of deaths from other causes could likely be related to snakebites. Mistakes regarding the recording of the clinical progress of the patient could be due to the notification form being filled in incorrectly or due to a lack of proper interaction and communication. For instance, if the patient died due to kidney failure or another complication, although it was due to envenomation, at the time, it may have been filed as “death from other causes.” It has been estimated that the reporting of mortality due to snakebites in Brazil is underreported by approximately 30%^15^. Recently published information reaffirms the importance of prevention measures and appropriate and timely treatment to avoid complications that can lead to death or sequelae^2^ ^-^ ^3^ ^,^ ^25^. 

This study has some strengths. It assessed surveillance data from a large time series and its limitations could have been minimized because of the large sample size analyzed. Moreover, it could be a data source for new studies by the surveillance system. Nonetheless, it is necessary to recognize the inherent limitations such as the use of secondary data fed by different sources and by different professionals involved in the reporting and, as a result, sometimes provided limited information regarding the variables of interest. Moreover, occasionally, the records are incomplete and have typographical errors. According to unpublished information, provided by the Ministry of Health, the administrators of SINAN mostly do not reclassify cases or clear up inconsistencies. There is also no procedure for capturing deaths that may have occurred after the patient was discharged because there is no follow-up after the report is prepared. The probabilistic linkage with other public health surveillance systems, such as the hospitalization data registry, could improve those limitations for future research. Moreover, there could be biases because of underreporting arising from the underutilization of health services. The fact that most patients, who are victims of these injuries, live in poor rural communities and do not have a strong political voice; consequently, snakebites are less of a priority for national public health programs and, as a result, snakebites fall into the category of major neglected tropical diseases^14^. The results presented here, however, are of great relevance because they provide information on the situation involving snakebites in the Brazilian Amazon during the study period. Furthermore, knowledge of the characteristics associated with severe outcomes in snakebites may help to identify patients who will require more intensive or longer follow-up and may provide the physician with counseling rationale for their patient’s possible prognosis.
